# Effects of *Bacillus subtilis* on growth performance, serum parameters, digestive enzyme, intestinal morphology, and colonic microbiota in piglets

**DOI:** 10.1186/s13568-020-01150-z

**Published:** 2020-12-02

**Authors:** Bo Deng, Jie Wu, Xiaohui Li, Cheng Zhang, Xiaoming Men, Ziwei Xu

**Affiliations:** 1 Institute of Animal Husbandry and Veterinary Science, Zhejiang Academy of Agricultural Sciences, Hangzhou, 310021 Zhejiang China; 2 Institute of Plant Protection and Microbiology, Zhejiang Academy of Agricultural Sciences, Hangzhou, 310021 Zhejiang China; 3 Hangzhou Zhengxing Animal Husbandry Co., Ltd., Hangzhou, 311301 Zhejiang China

**Keywords:** *Bacillus subtilis*, Piglets, Growth performance, Intestinal function, Serum parameters, Colonic microbiota

## Abstract

The present study was conducted to investigate effects of *Bacillus subtilis* on growth performance, serum parameters, digestive enzymes, intestinal morphology, and colonic microbiota in piglets. A total of 72 piglets were weighed and randomly allotted into three treatments (four replication pens per treatment with six piglets/pen) for a 28-day experiment. The dietary treatments were as follows: basal diet (control group, CTR), basal diet supplementation with antibiotic (antibiotic group, ABT), and basal diet supplementation with 0.1% *Bacillus subtilis* (probiotic group, PBT). The average daily gain of body weight increased in both the ABT and PBT groups, and dietary antibiotics decreased the feed:gain ratio (F:G), as compared to the CTR group (*P* < 0.05). Both ABT and PBT piglets had increased serum triglycerides and lipase, amylase, maltase activities and villus height:crypt depth ratio (V/C) in ileum (*P* < 0.05). The PBT group also showed an increase in serum glucose and villus height in the ileum (*P* < 0.05). Dietary antibiotics increased *Lactobacillus johnsonii*, as compared to the CTR group, but decreased bacterial diversity and increased *Escherichia coli*, as compared to the PBT group (*P* < 0.05). Piglets dietary with *B. subtilis* modulated the microbiota by increasing the abundance of *Firmicutes* (*L. johnsonii*, *L. reuteri*) and decreasing the abundance of *E. coli*, as compared to the control group (*P* < 0.05). These results indicate that dietary of *B. subtilis* improves growth performance and intestinal health and can be a promising alternative to antibiotics in piglets diet.

## Introduction

Environmental and nutritional stressors affect health and growth performance in all life phases of livestock production. Antibiotics have been included in feed formulations as growth promoter to improve feed efficiency and reduce pathogen infections for more than 50 years (Dibner and Richards 2005). However, there is increasing evidence that antibiotic abuse leads to bacterial resistance, which poses a huge risk for public health (Martin et al. 2015). The European Union banned antibiotics as growth promoter in 2006 and China will also prohibit prophylactic use of antibiotics in feed in 2020. Probiotics are considered one of potential alternative to antibiotics. The supplementation of probiotics have been widely reported to improve growth performance and intestinal immunity, enhance the intestinal epithelial barrier, and suppress pathogens (Resta-Lenert and Barrett 2006; Bermudez-Brito et al. 2012; Liao and Nyachoti 2017). *Bacillus subtilis* is “metabolically dormant and as close to indestructible as any cell found on earth; nonetheless, the spore retains the ability to revive almost immediately when nutrients return to the environment” (Driks 2002). Generally, *B. subtilis* is used in the spore form and can maintain stability in storage and be resistant to unfavorable environments during transit through the gastrointestinal tract. Numerous studies have demonstrated the beneficial effects of *B. subtilis* without safety concerns (Guo et al. 2006; Lee et al. 2014; Zhou et al. 2015). While not all strains are equally resistant to the environment in the gastrointestinal tract, and the pharmabiotic action of probiotics (including *Bacillus* spp.) from different strains is multi-factorial and strain-specific, some strains are more beneficial to the host than others (Weichselbaum 2009). In our previous research, we had demonstrated a strain of *B. subtilis* which improved intestinal function, reduced inflammation and developed microflora in LPS-induced acute inflammation rat (Deng et al. 2017). In the present study, we further evaluated the effect of *B. subtilis* on growth performance, serum parameters, digestive enzymes, intestinal morphology, and colonic microbiota in piglets.

## Materials and methods

### Source of probiotics

The *B. subtilis* bacterial strain (BF7658, CGMCC 1.240) was purchased from the China General Microbiological Culture Collection Center (CGMCC) and used in this study after being cultivated in Institute of Plant Protection and Microbiology, Zhejiang Academy of Agricultural Sciences. The strain containing *B. subtilis* at least 1 × 10^10^ CFU/g.

### Animals, diets, and experimental design

A total of 72 piglets of approximately 45 days of age and 14.06 ± 1.80 kg body weight (BW) (36 barrows and 36 gilts, Duroc × Large White × Landrace) were used in a 28-day feeding trail. All piglets were weighed and randomly assigned to three treatments and each treatment included four pens with six piglets/pen (three barrows and three gilts). The three treatments were: basal diet (control group, CTR), basal diet supplied with 40 mg/kg kitasamycin and 75 mg/kg chlorotetracycline (antibiotic group, ABT), and basal diet supplied with 0.1% *B. subtilis* (probiotic group, PBT). The basal diets were corn and soybean-based diets offered in meal form and were formulated to meet the specifications of growing piglets (Table 1).

**Table 1:** Composition and nutrition levels of the basal diet (as-fed basis)

Items	Content (%)
Ingredient	
Maize	45
Extrusion full fat soybean	13
Soybean meal	15
Extrusion maize	20
Fish meal	3
Dicalcium phosphate	1.1
Calcium carbonate	0.9
Sodium chloride	0.3
Premix^a^	1.7
Nutrient levels^b^	
Crude protein	18.56
DE (MJ/kg)	14.15
Crude fiber	2.72
Ether extract	4.39
Calcium	0.8
Total phosphorous	0.72
Digestible lysine	1.09
Digestible methionine	0.29
Digestible threonine	0.76
Digestible tryptophan	0.21

*DE* digestible energy

^a^Premix provided the following per kilogram of basal diet: Cu 20 mg, Zn 70 mg, Fe 100 mg, Mn 40 mg, Se 0.3 mg, I 0.4 mg. Vitamin A 7500 IU, vitamin D3 750 IU, vitamin E 25 IU, vitamin K3 2.0 mg, vitamin B1 1.88 mg, vitamin B2 3.75 mg, vitamin B6 2.19 mg, vitamin B12 0.025 mg, nicotinic acid 25 mg, d-pantothenic acid 15.6 mg, folic acid 2.0 mg, biotin 0.19 mg

^b^Nutrient levels were calculated values

Piglets were housed in pens with separated feeders and automatic, stainless steel, nipple drinkers. Feed and water were available ad libitum throughout the experimental period. A combination of daylight and artificial light was used and room temperature was controlled at 24 to 26 $^\circ {\text{C}}$. Piglets were observed at least twice per day for health status, and routine veterinary inspections were performed with additional visits upon the detection of ailments.

Piglets were weighted individually on day 0 and 28, and feed consumption was measured to calculate average daily gain (ADG), average daily feed intake (ADFI), and feed:gain ratio (F:G). On day 28, three healthy barrows and three healthy gilts from each treatment were selected to be euthanized with Zoletil (15 mg tiletamine/kg BW, 15 mg zolazepam/kg BW, intramuscular injection) and bled by exsanguinations.

### Sample collection

Blood samples were collected from the anterior vena into Eppendorf tubes. Serum samples were obtained after centrifugation at 3000 rpm for 10 min at 4 °C, and was stored at − 80 °C until analysis. Samples of the proximal duodenum, middle jejunum, and distal ileum were collected and washed with ice-cold physiological saline immediately. For histology, tissue samples were placed into 10% (v/v) neutral buffered formalin. For enzyme activity analysis, mucosal samples were scraped off each intestinal segment onto a glass slide and snap frozen into liquid nitrogen. Colonic digesta were collected into Eppendorf tubes and immediately snap frozen in liquid nitrogen for 16S high-throughput sequencing.

### Intestinal morphology

After fixation in neutral buffered formalin for 24 h, tissues of each intestine segment were embedded in paraffin. A section of 0.5 cm thickness was stained with hematoxylin and eosin (HE) to analyze villus height and crypt depth. Images were acquired using an Olympus DP-71 digital camera (OLYMPUS Corporation, Shinjuku, Tokyo, Japan). Mean villous height and mean crypt depth were determined using an image processing system equipped with Image-ProPlus 5.0 (Media Cybernetics, Maryland, USA). The detail method was based on previously published methods (Lee et al. 2014).

### Serum biochemical parameters and mucosal digestive enzymes

The concentration of serum biochemical parameters: glucose, triglyceride (TG), total cholesterol (TC), high density fatty acid (HDLC) and low density fatty acid (LDLC) were measured by commercial assay kits (Nanjing Jiancheng Bio-Engineering Institute, Nanjing, China) according to the manufacturer’s instructions (Ding et al. 2020).

Approximately 0.5 g of frozen intestinal mucosal samples were homogenized in 4.5 mL ice-cold 0.9% NaCl solution by a homogenizer. Then the homogenates were centrifuged at 4 $^\circ {\text{C}}$ for 15 min at 4000 rpm to obtain the supernatant. The activities of digestive enzymes (trypsase, lipase, amylase, sucrase, lactase, and maltase) in the supernatants were measured by commercial assay kits (Nanjing Jiancheng Bio-Engineering Institute, Nanjing, China) according to the manufacturer’s instructions (Rajput et al. 2013; Huo et al. 2017).

### High-throughput sequencing

Total DNA from colon digesta was extracted using a MicroElute Genomic DNA kit (D3096-01, Omega, Inc., USA). The V3–V4 fragment of 16S rRNA was amplified using the total DNA as a template and the conserved primers are 319F (5ʹ-ACTCCTACGGGAGGCAGCAG-3ʹ) and 806R (5ʹ-GGACTACHVGG GTWTCTAAT-3ʹ) (He et al. 2019). The specific steps are shown in Additional file 1. All sequences generated in this study have been submitted to the NCBI Sequence Read Archive (https://www.ncbi.nlm.nih.gov/sar) under accession number SRA: PRJNA665361.

### Statistical analyses

The analyses of growth performance, serum parameters, digestive enzymes, and intestinal morphology, relative abundance of phylum and species between groups were performed by one-way ANOVA with line segment detector (LSD) using the SPSS 22.0 software (SPSS Inc., Chicago, IL, USA). Alpha diversity and taxonomic community assessments were performed using Qiime 1.7.0. Principal coordinates analysis (PCoA) and nonmetric multidimensional scale analysis (NMDS) plots were generated using weighted UniFrac metrics. The experiment unit of growth performance is pen and experiment unit of other index is individual animal. All data are shown as the mean ± standard error of mean (SEM). Statistical significance was considered at *P* < 0.05.

## Results

### Growth performance

Supplementation of antibiotics increased final BW and ADG, and decreased F:G (*P* < 0.05; Table 2). Piglets supplemented with *B. subtilis* also showed positive effects in growth performance, with increased final BW and ADG, as compared to the CTR group (*P* < 0.05). No significant differences were observed between the ABT and PBT group (*P* > 0.05).

**Table 2:** Effects of dietary *B. subtilis* supplementation on growth performance of piglets

Items	CTR^c^	ABT^d^	PBT^e^	SEM^f^	*P-*value
Initial body weight (kg)	14.07	14.05	14.07	0.005	0.14
Final body weight (kg)	28.27^b^	31.22^a^	31.15^a^	0.53	0.002
ADG (kg)	0.51^b^	0.61^a^	0.61^a^	0.12	0.002
ADFI (kg)	1.00	1.11	1.13	0.03	0.08
F:G	1.97^a^	1.81^b^	1.84^a, b^	0.03	0.04

*ADG* average daily gain, *ADFI* average daily feed intake, *F:G* feed:gain ratio

^a, b^Value within a row with different superscripts were considered to be significant when P < 0.05 (n = 4 per treatment)

^c^*CTR* control group (basal diet); the same as below

^d^*ABT* antibiotic group (basal diet supplied with 40 mg/kg kitasamycin and 75 mg/kg chlorotetracycline); the same as below

^e^*PBT* probiotic group (basal diet supplied with 0.1% *B. subtilis*); the same as below

^f^*SEM* standard error of the mean; the same as below

### Serum parameters

The analysis of serum parameters showed that antibiotic treatment markedly increased serum triglyceride and cholesterol (*P* < 0.05, Table 3). *B. subtilis* supplementation increased serum glucose and triglyceride (*P* < 0.05), while dietary supplementation of antibiotic or probiotics had no effect on serum HDLC and LDLC levels (*P* > 0.05).

**Table 3:** Effects of dietary *B. subtilis* supplementation on serum parameters of piglets

Items (mmol/L)	CTR	ABT	PBT	SEM	*P-*value
Glucose	5.56^b^	6.48^a^	8.57^a^	0.51	0.005
Triglyceride	0.27^b^	0.47^a^	0.48^a^	0.04	0.009
Cholesterol	2.93^b^	3.65^a^	3.00^b^	0.22	0.035
HDLC	1.03	0.99	1.12	0.07	0.15
LDLC	1.36	1.47	1.46	0.09	0.67

*HDLC* high density fatty acid, *LDLC* low density fatty acid

^a, b^Value within a row with different superscripts were considered to be significant when P < 0.05 (n = 6 per treatment), the same as Tables 4, 5

### Mucosal digestive enzymes

Activity of amylase and lipase in the ileum were significantly increased in both ABT and PBT groups (*P* < 0.05) (Table 4). In addition, dietary supplementation of *B. subtilis* also improved lactase and maltase activity in the ileum, as compared to both the CTR and ABT groups (*P* < 0.05).

**Table 4:** Effects of dietary *B. subtilis* supplementation on mucosal digestive enzyme of piglets

	Items	CTR	ABT	PBT	SEM	*P-*value
Duodenum	Trypsase (U/mg protein)	49.99	56.71	66.09	4.03	0.11
	Lipase (U/g protein)	1.93	1.85	2.54	0.17	0.20
	Amylase (U/mg protein)	0.61	0.54	1.25	0.12	0.05
	Sucrase (U/mg protein)	8.64	7.89	7.70	0.69	0.84
	Lactase (U/mg protein)	11.56	11.83	10.43	0.73	0.39
	Maltase (U/mg protein)	169.30	154.25	169.82	11.92	0.29
Jejunum	Trypsase (U/mg protein)	85.28	99.40	102.95	7.79	0.56
	Lipase (U/g protein)	3.40	3.69	3.64	0.24	0.76
	Amylase (U/mg protein)	1.36	1.30	1.40	0.09	0.89
	Sucrase (U/mg protein)	80.59	74.47	70.10	6.38	0.82
	Lactase (U/mg protein)	20.22	26.26	26.88	1.81	0.14
	Maltase (U/mg protein)	165.06	177.27	153.61	11.17	0.29
Ileum	Trypsase (U/mg protein)	216.71	162.73	222.37	14.79	0.16
	Lipase (U/g protein)	3.71^b^	5.32^a^	4.92^a^	0.4	0.004
	Amylase (U/mg protein)	0.44^b^	1.66^a^	2.25^a^	0.23	0.014
	Sucrase (U/mg protein)	65.04	64.86	74.56	6.1	0.85
	Lactase (U/mg protein)	4.08^b^	4.21^b^	6.19^a^	0.4	0.005
	Maltase (U/mg protein)	111.94^c^	142.55^b^	185.51^a^	11.88	0.002

### Intestinal morphology

The villus height/crypt depth ratio (V/C) in ileum markedly increase with dietary supplementation of antibiotics or *B. subtilis*. Also, villus height was significantly improved in the ileum of piglets in the PBT group as compared to CTR group (*P* < 0.05, Table 5).

**Table 5:** Effects of dietary *B. subtilis* supplementation on intestinal morphology of piglets

	Items	CTR	ABT	PBT	SEM	*P*-value
Duodenum	Villus height	197.07	180.33	197.82	11.64	0.49
	Crypt depth	230.76	216.29	203.30	13.28	0.53
	V/C	0.92	0.84	0.98	0.06	0.16
Jejunum	Villus height	169.19	156.93	170.22	10.25	0.78
	Crypt depth	154.34	144.36	163.07	9.55	0.46
	V/C	1.11	1.12	1.06	0.07	0.71
Ileum	Villus height	106.34^b^	130.55^a, b^	141.26^a^	9.27	0.04
	Crypt depth	148.59	129.42	140.11	9.8	0.41
	V/C	0.74^b^	1.07^a^	1.04^a^	0.08	0.04

*V/C* villus height/crypt depth ratio

### Colonic microbiota

**Fig. 1:**
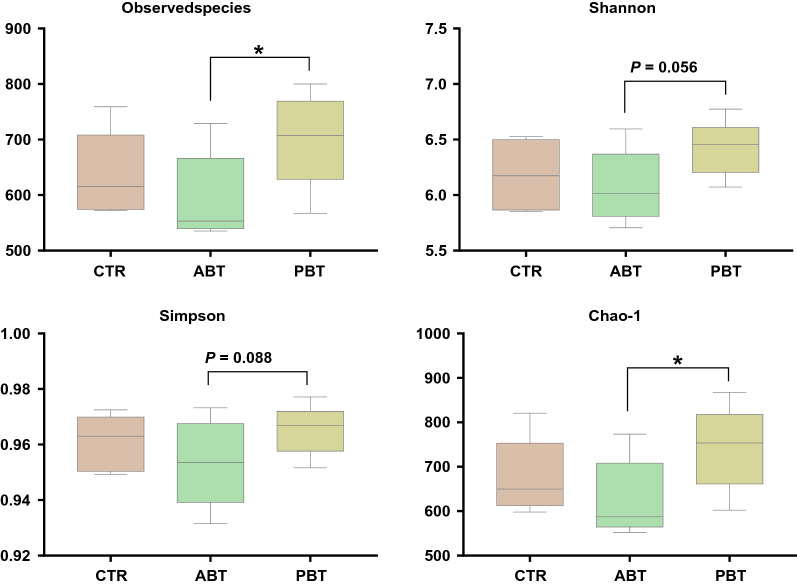
Effects of dietary *B. subtilis* supplementation on colonic microbial community diversity of piglets. Data are expressed as mean ± SEM. * Were considered to be significant when P < 0.05

Dietary supplementation of *B. subtilis* significantly increased microbiota and the Chao-1 index (*P* < 0.05), and tended to increase the Shannon index (*P* = 0.056) and the Simpson index (*P* = 0.088) as compared to the ABT group (Fig. 1). No differences were observed between the CTR and PBT groups (*P* > 0.05).

**Fig. 2:**
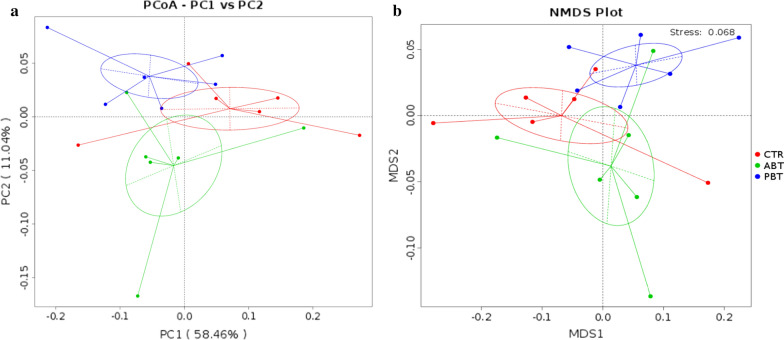
Effects of dietary *B. subtilis* supplementation on colonic microbial community structures of piglets. **a** Represents PCoA analyze based on the weighted UniFrac distance matrixes, **b** represents NMDS analyze based on the weighted UniFrac distance matrixes

To describe the gut microbiota of piglets, distance matrices were calculated by weighted UniFrac and visualized via PCoA and NMDS (Fig. 2). Piglets on the same feed tended to cluster together and were separate from other groups. PBT samples were mostly separated from ABT samples.

**Fig. 3:**
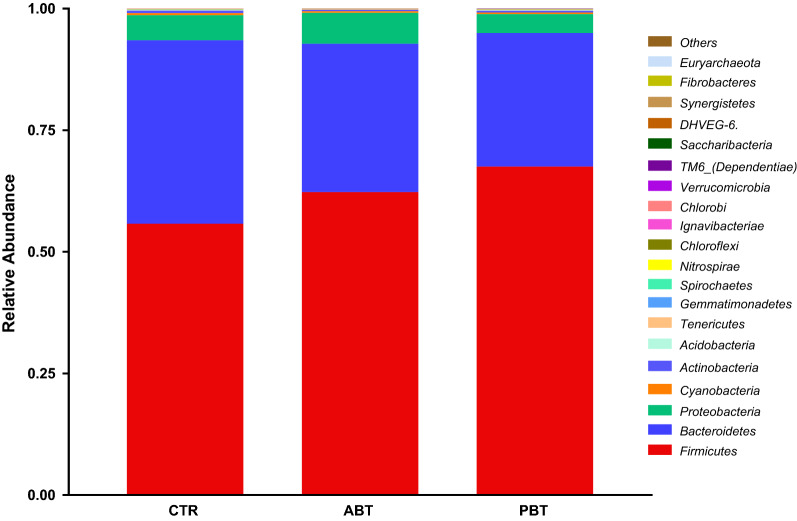
Effects of dietary *B. subtilis* supplementation on colonic microbiota composition at phylum level with the relative abundance within the top 20 of piglets

Phylum distributions show that the colonic microbiomes were dominated by *Firmicutes* and *Bacteroidetes*, which comprised 90% of the total bacteria in piglets (Fig. 3). Dietary supplementation of antibiotics showed limited effects on these two bacterial populations (*Firmicutes* CTR 55.50% vs ABT 62.29%, *Bacteroidetes* CTR 38.84% vs ABT 30.47%). Dietary supplementation of *B. subtilis* increased *Firmicutes* (CTR 55.50% vs PBT 67.54%, *P* < 0.05), and tended to decrease the abundance of *Bacteroidetes* (CTR 38.84% vs PBT 27.42%,* P* = 0.059).

**Fig. 4:**
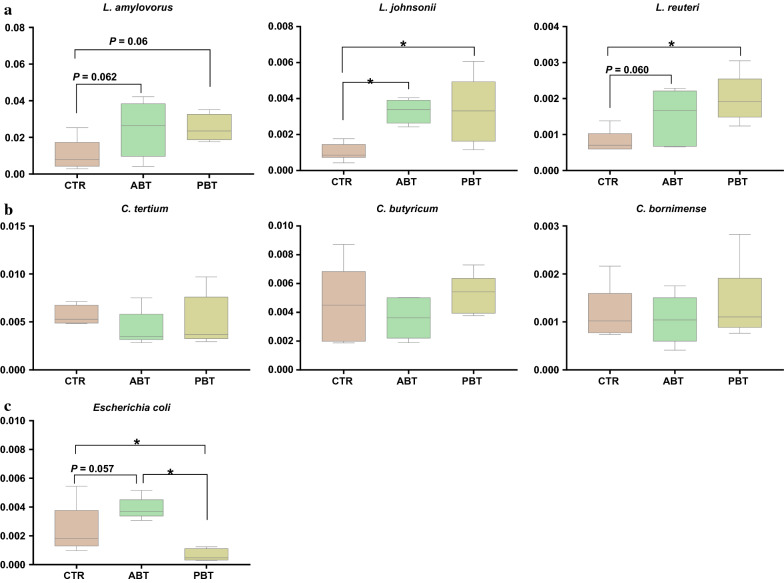
Effects of dietary *B. subtilis* supplementation on colonic relative abundance (%) of *Lactobacillus* spp. (**a**), *Clostridium* spp. (**b**), and *Escherichia coli* (**c**) within the top 25 species levels of piglets. Data are expressed as mean ± SEM. * Were considered to be significant when P < 0.05

At species level, we focused attention on *Lactobacillus* spp. (a), *Clostridium* spp. (b), and *E. coli* (c) within the top 25 species (Fig. 4). The results showed that dietary supplementation of antibiotics markedly increased the abundance of *L. johnsonii *(*P* < 0.05), and tended to increase the abundance of *L. amylovorus* (P = 0.062) and *L. reuteri *(*P* = 0.06)*.* Antibiotics also tended to increase the abundance of *E. coli*, as compared to the CTR group (*P* = 0.057)*.* Dietary supplementation of *B. subtilis* significantly increased the level of *L. johnsonii* and *L. reuteri *(*P* < 0.05) and tended to increase *L. amylovorus* (*P* = 0.06)*.* Moreover, *B. subtilis* decreased *E. coli*, as compared to both the CTR and ABT groups (*P* < 0.05). No differences were noted in the abundance of the three *Clostridium* spp. between all groups.

## Discussion

*Bacillus subtilis* is one of most commonly used probiotics in livestock feed, but previous studies have indicated contrasting results in livestock production, which might be due to different hosts or even the use of probiotics of different species (Kritas and Morrison 2005; Lee et al. 2014). Our previous study isolated a strain of *B. subtilis* and demonstrated its effectiveness in LPS-induced acute inflammation in rats. In the present study, *B. subtilis* was used in piglets to determine whether it could effectively replace antibiotics.

Improved growth performance in piglets supplemented with *B. subtilis* or antibiotics was observed in the present study, and antibiotics showed a higher feed efficiency. The results agreed with Lee et al.’s (2014) research, who reported a linear improvement in both ADG and ADFI with increasing *B. subtilis* in the diet (0.15, 0.3, and 0.45 mg/kg). Gracia et al. (2004) also found that adding 0.04% of a probiotic mixture including *B. subtilis* and *B. licheniformis* could benefit ADG and ADFI during the pre-starter period and the overall pre-starter-finishing period. There are contradicting results, however. Kritas and morrison’s (2005) research failed to demonstrate any effects of *B. subtilis* and *B. licheniformis* inclusion on growth performance in piglets. Jørgensen et al. (2016) even noted that feeding 400 mg/kg of a *Bacillus*-based probiotic decreased ADG and increased F:G in the grower period. The variability of results on growth performance may be associated with dose level, animal age, strain source, host health status or even the administration strategies (Wang et al. 2009; Lee et al. 2014).

We measured the serum parameters including glucose, TG, TC, HDLC, and LDLC to evaluate the nutrition levels in piglets. Significant improvements in glucose and TG were observed in piglets with supplementary *B. subtilis*. Ding et al. (2020) also demonstrated that basal diets supplemented with 0.05% *B. subtilis* had higher plasma glucose and triglycerides on day 28 and day 42, and higher α-amylase and LDLC on day 28 in nursery piglets. Rajput et al.’s (2013) research showed that *B. subtilis* decreased the content of TG in broilers. In mice fed high fat diets, decreased lipid profiles were also noted by Zouari et al. (2016) when *B. subtilis* SPB1 was included in the feed. The differences may be due to energy intake levels and growth stage. In the starter period of pig growth, especially during weaning, glucose and TG are major sources of energy but energy intake commonly cannot meet the requirement (Bruininx et al. 2001). In this case, the glucose and TG provided by *B. subtilis* would be beneficial to fast and healthy growth of piglets.

Digestive enzymes are important to hydrolyze food into smaller and absorbable components which directly influence the nutrient digestibility and growth performance. Intestinal, disaccharidase-specific activities are also used to measure intestinal health and maturity in response to dietary factors (Pi et al. 2014). In the present study, dietary supplementation of *B. subtilis* improved tryptase activity in the duodenum and lipase, amylase, lactase, and maltase activity in the ileum. The results indicated that *B. subtilis* might support the action of lipid, protein, and carbohydrate metabolism and promote the maturity of mucosa in piglets. Our findings are in agreement with Huo et al.’s (2017) research, who illustrated that 0.1% *B. subtilis* Z-27 markedly increased the activity of acid protease, lipase, α-amylase, and cellulose in the duodenum, jejunum and ileum of weaned piglets. Similarly, Rajput et al. (2012) reported that dietary supplementation of 1 × 10^8^ CFU/kg *B. subtilis* for 35 days increased amylase and trypsin activity in Shaoxin duck.

Small intestinal morphology is a crucial factor in the maintenance of normal intestinal functions. In the current study, dietary *B. subtilis* markedly increased villus height in the ileum and tended to increase V/C in the ileum. The positive changes in intestinal morphology indicated improved growth and a better assimilation in the gut. We did not measure nutrient digestibility, but the results of serum glucose, lipid metabolites, and digestive enzymes support the speculation. The finding is in agreement with Lee et al. (2014) who noted a positive effect on villus height and V/C in the duodenum, jejunum, and ileum in pigs fed with 0.45% *B. subtilis* LS 1–2. Kim et al. (2012) also demonstrated that supplementation of a multi-microbe probiotic product containing *B. subtilis* in broiler diets improved villus height and V/C in the duodenum and ileum at day 35. Other studies have demonstrated that the intestinal morphology was not altered by *B. subtilis*. Choi et al. (2011) demonstrated that multi-microbe probiotics containing *B. subtilis* showed limited effects on villus height and crypt depth in different intestinal segments. In *E.* coli K88-challenged pigs, dietary supplementation with a *B. subtilis-*based microbial also failed to improve intestinal morphology (Bhandari et al. 2008). The different results may due to the supplementation level and healthy status of the host.

The bacterial composition and diversity of gut microbes is very dynamic and can be influenced by several factors. Bhandari et al. (2008) noted that *B. subtilis* markedly increased bacterial richness and divty in *E.* coli K88-challenged piglets. Another study by Hu et al. (2014) revealed that weaned piglets treated with *B. subtilis* KN-42 showed the highest bacterial diversity by measuring the number of identifiable DGGE bands. Our recent research also found that *B. subtilis* increased the microbiota and Chao-1 index in rats (Deng et al. 2017). The increased bacterial diversity means more stable bacterial communities which increase the host’s ability to respond to perturbations (McCann 2000). *Firmicutes* and *Bacteroidetes* are two dominant bacterial phyla in pigs and humans and are linked processes impacting host health, such as fat metabolism and carbohydrate fermentation (Lee et al. 2009). Cui et al. (2013) noted that dietary of *B. subtilis* increased the ratio of *Firmicutes* to *Bacteroidetes* in a long term study of pigs from 10 to 110 kg. In the present study, we also observed an increase of the abundance of *Firmicutes* and a decrease in *Bacteroidetes* in *B. subtilis*-treated piglets. The alteration of the *Firmicutes*/*Bacteroidetes* ratio suggests that *B. subtilis* might promote the metabolic activities and fermentation of complex plant-based diets and tend to deposit fat (Cui et al. 2013). A study by Guo et al. (2008) also revealed that a higher *Firmicutes/Bacteroidetes* ratio normally corresponds to an increased BW, which is consistent with our study. The ratio of *Firmicutes* to *Bacteroidetes* showed a strong negative correlation to pathogens in the gut and a positive correlation to intestinal short chain fatty acids (Molist et al. 2012), which might reduce infection and responses to unfavorable environments (Mulder et al. 2009).

*Lactobacillus* spp. are considered as beneficial microorganisms that increase intestinal function, improve immune system, and modify intestinal microflora, thereby improving overall health of the host. In the present study, *B. subtilis* showed a positive effect on the proliferation of the three *Lactobacillus* spp. including *L. johnsonii*, *L. reuteri*, and *L. amylovorus.* The result was similar to those of previous studies. Guo et al. (2006) reported that *B. subtilis* MA139 with 2.2 × 10^5^ CFU/g supplementation for piglets to 28 days markedly increased *Lactobacillus* spp. in the feces. A previous study by Wang et al. (2019) also showed that 0.1% *B. subtilis* (1 × 10^9^ CFU/kg) promoted the proliferation of *Lactobacillus* in feces of piglets, while the effect was absence in the lower viable count (1 × 10^8^ CFU/kg). We also noted that *B. subtilis* suppressed the growth of *Escherichia coli*. *E. coli* is a major pathogen causing diarrhea in piglets. Decreased *E. coli* suggested that *B. subtilis* could effectively prevent diarrhea in piglets (Fairbrother et al. 2005). The results concur with Tsukahara et al.’s (2013) study, who found that oral administration of *B. subtilis* DB9011 decreased growth of Shiga toxin 2e-producing *E. coli* in piglets. Another study by Giang et al. (2011) also revealed that dietary supplementation of *Bacillus* showed positive effects on decreasing *E. coli* counts in feces and, combined with *Saccharomyces* and lactic acid bacteria, the effect was reinforced. The potential mechanism of antimicrobial effects might be associated with the metabolites produced by *B. subtilis*, such as lipopeptides and aminocumaim A.

Kitasamycin and chlorotetracycline were used as an antibiotic combination to evaluate the *B. subtilis* here in used. The results revealed that supplementation with *B. subtilis* showed similar effects to antibiotics in most indexes including growth performance, intestinal morphology, or even better on some aspects, such as digestive enzyme activity. The use of probiotics as an alternative to antibiotics was also shown in previous studies by using single or multi-probiotic products (Shen et al. 2009; Choi et al. 2011). As a growth promoter, it remains controversial as to whether probiotics would be effective as antibiotics. Afsharmanesh et al. (2013) noted that growth performance was significantly lower in broiler chickens fed 8 × 10^5^ CFU/kg *B. subtilis* in comparison to antibiotics, even though probiotics produced a better intestinal morphology. The reasons for this are complex, and may be related to host health state, strain of probiotic, dosage of antibiotic, and the management environment. In the present study, room temperature was about 24 °C and initial BW was about 14 kg, which suggested a favorable environment for growth and strong resistance to disease, which might mask the effects of the antibiotic. Intestinally, we noted that antibiotics and probiotics showed different effects on gut microbes. Although both antibiotics and *B. subtilis* increased *Lactobacillus* spp., antibiotics decreased colonic bacterial diversity and increased *E. coli*, as compared to the probiotic group. Similar results were also shown in Dethlefsen et al.’s (2008) study, who found that 5 days of treatment with Ciprofloxacin decreased taxonomic richness within days of initial exposure. Looft et al. (2012) observed that swine fed ASP250 containing chlortetracycline, sulfamethazine, and penicillin increased *E. coli* population at 14 days, showing a 20- to 100-fold greater *E. coli* abundance in medicated than non-medicated swine. The different action on gut microbes could be an advantage of probiotics.

In conclusion, this study demonstrated that dietary of *B. subtilis* was effective in improving growth performance, digestive enzyme activity, and intestinal morphology. Furthermore, *B. subtilis* also increased bacterial diversity, increased *Lactobacillus* spp., and decreased *E. coli* in piglets. These results suggest that *B. subtilis* is a promising alternative to antibiotics in piglet feed. However we should note that the piglets used in this study is relatively big but not just weaned, more experiments with different conditions such as different growth periods or different feeding environment need to be carried out to verify the efficiency of *B. subtilis*. And the underlying mechanisms of how this *B. subtilis* strain works should be further assessed by analyzing its metabolites.

## Supplementary information

**Additional file 1.** Methods for Amplification of V3–V4 region

## Data Availability

The datasets used or analyzed during the current study are available from the corresponding author on reasonable request.

## Abbreviations

CTR: Control group
ABT: Antibiotic group
PBT: Probiotic group
*B. subtilis*: *Bacillus subtilis*
*E. coli*: *Escherichia coli*
F:G: Feed:gain ratio
BW: Body weight
ADG: Average daily gain
ADFI: Average daily feed intake
HE: Hematoxylin and eosin
TG: Triglyceride
V/C: Villus height:crypt depth ratio
PCoA: Principal coordinates analysis
NMDS: Nonmetric multidimensional scale analysis
