# Supplementary material for: Effects of Bacillus subtilis on growth performance, serum parameters, digestive enzyme, intestinal morphology, and colonic microbiota in piglets
Source: AMB Express. 2020 Dec 2;10:212. doi: 10.1186/s13568-020-01150-z (PMC7710768; doi:10.1186/s13568-020-01150-z)
Supplement: Supplementary file 1 — Additional file 1. Methods for Amplification of V3–V4 region [file 13568_2020_1150_MOESM1_ESM.docx]

**Amplification of V3–V4 region**

DNA from different samples was extracted using the MicroElute Genomic DNA Kit (D3096-01, Omega, Inc., USA) according to manufacturer’s instructions. The reagent which was designed to uncover DNA from trace amounts of sample has been shown to be effective for the preparation of DNA of most bacteria. Sample blanks consisted of unused swabs processed through DNA extraction and tested to contain no 16S amplicons. The total DNA was eluted in 50 µl of Elution buffer by a modification of the procedure described by manufacturer (QIAGEN) and stored at -80°C until measurement.

Using the total DNA as a template and the primer (319F 5'-ACTCCTACGGGAGGC- AGCAG-3'; 806R 5'-GGACTACHVGGGTWTCTAAT-3'), we amplified the V3–V4 region of the bacterial 16S rRNA. All reactions were carried out in 25µl(total volume) mixtures containing approximately 25 ng of genomic DNA extract, 12.5µl PCR Premix, 2.5µl of each primer, and PCR-grade water to adjust the volume. PCR reactions were performed in a Master cycler gradient thermocycler (Eppendorf, Hamburg, Germany) set to the following conditions: initial denaturation at 98℃for 30 seconds; 35cycles of denaturation at 98 ℃for 10 seconds, annealing at 54 ℃/52 ℃for 30 seconds, and extension at 72 ℃ for 45 seconds; and then final extension at 72 ℃for 10 minutes. The PCR products were confirmed with 2% agarose gel electrophoresis. Throughout the DNA extraction process, ultrapure water, instead of a sample solution, was used to exclude the possibility of false-positive PCR results as a negative control. The PCR products were normalized by AxyPrep TM Mag PCR Normalizer (Axygen Biosciences, Union City, CA, USA), which allowed for the skipping of the quantifications step regardless of the PCR volume submitted for sequencing. The amplicon pools were prepared for sequencing with AMPure XT beads (Beckman Coulter Genomics, Danvers, MA, USA) and the size and quantity of the amplicon library were assessed on the LabChip GX (Perkin Elmer, Waltham, MA, USA) and with the Library Quantification Kit for Illumina (Kapa Biosciences, Woburn, MA, USA), respectively. PhiX Control library (v3) (Illumina) was combined with the amplicon library (expected at 30%). The library was clustered to a density of approximately 570 K/mm2. The libraries were sequenced either on 300PE MiSeq runs and one library was sequenced with both protocols using the standard Illumina sequencing primers, eliminating the need for a third (or fourth) index read.
